# Identification, Expression Profiling, Microbial Binding, and Agglutination Analyses of Two Cathepsin B Genes in Black Rockfish (*Sebastes schlegelii*)

**DOI:** 10.3390/md23050213

**Published:** 2025-05-18

**Authors:** Xinghua Zhuang, Xingchun Li, Wenpeng Li, Xuan Xu, Fengjun Lin, Yiying Liu, Chonghui Chen, Xiaoxu Zhang, Pei Zhang, Chao Li, Qiang Fu

**Affiliations:** 1 School of Marine Science and Engineering, Qingdao Agricultural University, Qingdao 266109, China; 13210811286@163.com (X.Z.); 18953659537@163.com (X.L.); xuxuan989826@126.com (X.X.); 17664080757@163.com (F.L.); 15263348506@163.com (Y.L.); c13325030859@163.com (C.C.); 17664020172@163.com (X.Z.); zhangpei202153@163.com (P.Z.); chaoli@qau.edu.cn (C.L.); 2 Haidu College, Qingdao Agricultural University, Laiyang 265200, China; liwenpeng2008@163.com

**Keywords:** cathepsin, teleost, expression pattern, innate immunity

## Abstract

As a lysosomal cysteine protease of the papain subfamily, cathepsin B (CTSB) is characterized by its innate immune functions and hydrolytic activity. However, the functions of CTSB in the immune responses of teleosts remain to be clarified. In this study, two CTSB genes in *S. schlegelii*, *Ss*CTSBa and *Ss*CTSBb, were identified. Both *Ss*CTSBa and *Ss*CTSBb are composed of a 993 bp ORF encoding 330 amino acids. It was found in a phylogeny analysis that both genes form monophyletic clades with their orthologous counterparts of Honeycomb rockfish (*Sebastes umbrosus*). A synteny analysis indicated that the CTSB homologues were comparatively conserved during vertebrate evolution. Additionally, quantitative real-time PCR revealed the ubiquitous mRNA expression of *Ss*CTSBa and *Ss*CTSBb in all of the examined tissues, and substantially differential expression patterns could be observed following *Aeromonas salmonicida* infection. A subcellular localization analysis demonstrated that the distribution of *Ss*CTSBa and *Ss*CTSBb was mainly in the cytoplasm. Moreover, r*Ss*CTSBa and r*Ss*CTSBb showed strong binding to Poly(I:C) and exhibited diverse agglutination effects on different bacteria. Overall, these findings suggest that the CTSB genes in black rockfish might show essential functions in the host defense of teleosts against bacterial infections, providing valuable insights for further investigations into the immune mechanism of teleost CTSB.

## 1. Introduction

Cathepsins are considered to be the primary members of the papain family, and are referred to as proteolytic enzymes that exist in animals and plants [1]. Additionally, cathepsins can be classified into three groups based on their conserved amino acid residues: cysteine proteases (cathepsins B, C, F, H, K, L, O, S, T, U, V, W, X, and Z) [2], serine proteases (cathepsins A and G), and aspartic proteases (cathepsins D and E) [3]. Cysteine proteases are primarily from the C1 peptidase family, otherwise known as the papain family. In addition, cathepsins B, C, F, H, L, O, and Z are universally expressed across various tissues, whereas cathepsins K, W, and X exhibit cell-specific or tissue-specific expression patterns according to their tissue distribution [4]. Moreover, cysteine cathepsins can be further categorized into the B-like subfamily (cathepsins B, C, O, and X) and the L-like subfamily (cathepsins L, V, K, S, W, F, and H), depending on the sequence length, similarity, and structure of the pro-region [5,6].

Cathepsin B belongs to the B-like subfamily of lysosomal proteases, which was initially reported in mice [7]. It has been extensively studied for its involvement in various proteolytic process such as antigen presentation [8], antigen degradation [9], and apoptosis [10]. In mammals, CTSB is significantly expressed in immune cells, including monocytes and macrophages [9]. In addition, CTSB was reported to be involved in numerous physiological processes linked to diseases, including the inflammatory response [11], tumor invasion and metastasis [12], and Alzheimer’s disease [13]. Recently, the roles of CTSB in fish species’ innate immunity have received more attention [14,15,16], and the homologues of numerous fish species have been cloned and characterized, such as miiuy croaker (*Miichthys miiuy*) [17], channel catfish (*Ictalurus punctatus*) [18], large yellow croaker (*Pseudosciaena crocea*) [8], and olive flounder (*Paralichthys olivaceus*) [19]. In Japanese flounder (*Paralichthys olivaceus*), CTSB is markedly elevated in the spleen, kidneys, gills, and intestine with LPS and viral infection [20]. In golden pompano (*Trachinotus ovatus*), CTSB showed an obvious increasing trend in the liver, spleen, and head kidney under challenge by *Edwardsiella tarda* [21]. Overall, these findings suggest that CTSB might be involved in anti-microbial immune responses. Nevertheless, the specific immunological participation of CTSB in immune responses to bacterial infection remains to be explored.

As the major commercial species in mariculture, black rockfish (*S. schlegelii*) have been widely cultivated in China, South Korea, and Japan. However, persistent bacterial problems have caused serious losses in the aquaculture industry, particularly with *A. salmonicida* infection [22]. In addition, although the functions of the genome and virulence factors of *A. salmonicida* [23,24] have been identified, the interaction between this bacterium and host tissues in infected black rockfish remains to be clarified. Furthermore, a comprehensive investigation into the innate immune responses of black rockfish to pathogen infections is urgently required to develop effective strategies for disease prevention and control in aquaculture. The cathepsin superfamily in the *S. schlegelii* genome has been systematically analyzed in previous studies, which has indicated the important roles of bacterial infections [25]. However, the immune role of cathepsins requires further investigation. Two homologues of cathepsin B from black rockfish have been systematically characterized in this study, and the expression patterns have been deeply evaluated in multiple tissues of healthy fish and after infection. Additionally, the subcellular localization of these homologues has been investigated in this study. Recombinant proteins were obtained using a prokaryotic expression system, enabling the determination of their binding ability as well as agglutination capacity towards microbial ligands or bacteria in vitro. Overall, this research could provide a foundation for further exploration into the immune functions of CTSB.

## 2. Results

### 2.1. Identification of S. schlegelii CTSB

The full-length cDNA of *Ss*CTSBa (GenBank Accession: PQ683368) was composed of a 993 bp ORF that encoded a putative protein consisting of 330 amino acid residues with a predicted molecular mass of 36.06 kDa, a theoretical isoelectric point of 5.38, an aliphatic index of 69.42, and an instability index of 32.27. The GRAVY score was −0.260, with three cysteine protease active sites. Additionally, a total of 37 negatively and 27 positively charged residues were observed in this study (Table 1). In contrast, the full-length cDNA of *Ss*CTSBb (GenBank Accession: PQ683369) contained a 993 bp ORF that encoded 330 amino acid residues with a calculated molecular mass of 36.06 kDa and a theoretical isoelectric point of 5.20, in which the aliphatic and instability indexes were 70.94 and 31.46, respectively. The GRAVY score was −0.309 with three cysteine proteases active sites. In addition, a total of 37 negatively charged and 24 positively charged residues were observed. According to the amino acid sequence alignment, *Ss*CTSBa and *Ss*CTSBb shared overall identities at a relatively high level (97.57% and 99.09%, respectively) with honeycomb rockfish cathepsin B homologues. In contrast, *Ss*CTSBa and *Ss*CTSBb shared relatively low sequence identity (70.18% and 66.77%, respectively) with cathepsin B homologues in humans (Figure S1).

### 2.2. Characteristics of S. schlegelii CTSB Genes

**Figure 1:**
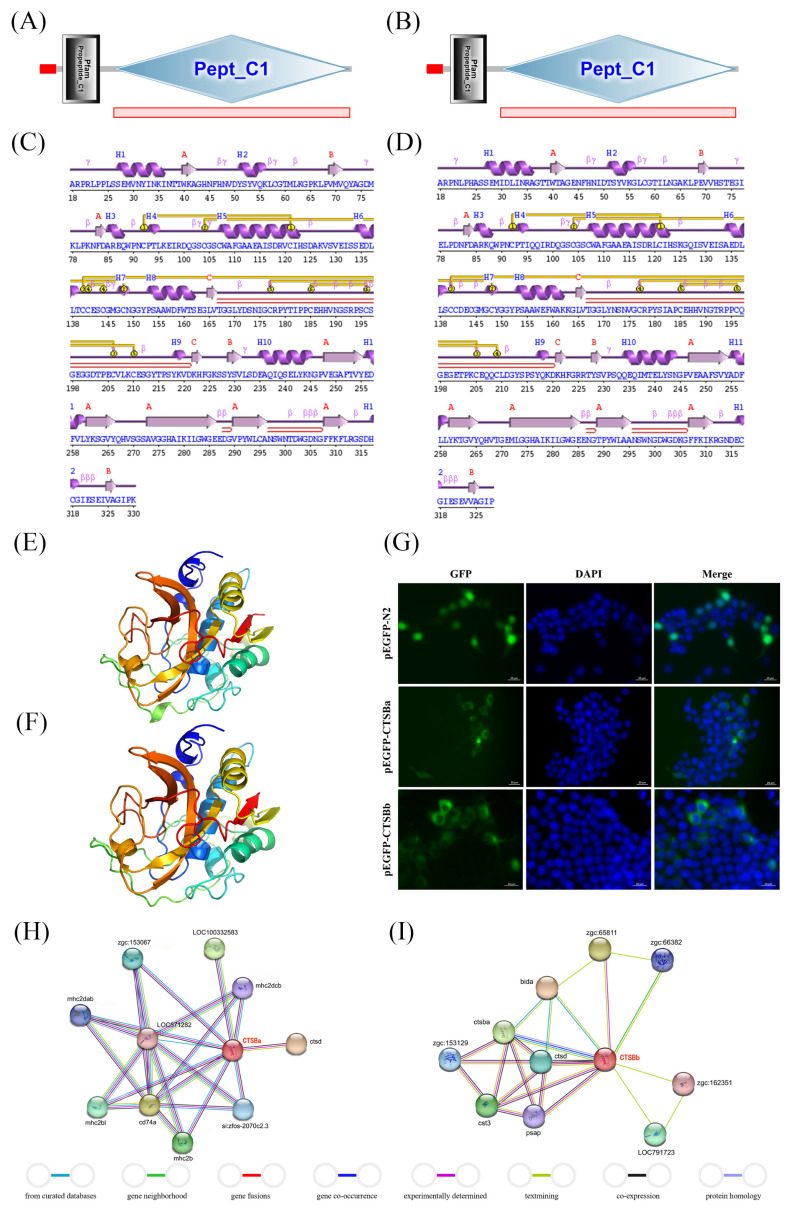
Bioinformatics analysis of *Ss*CTSBa and *Ss*CTSBb. (**A**,**B**) Sequence analysis of *Ss*CTSBa and *Ss*CTSBb. The amino acid sequences of signal peptide, Pfam Propeptide_c1, and Pept_C1 domain were analyzed by SMART. (**C**,**D**) Secondary structures of *Ss*CTSBa and *Ss*CTSBb were predicted using PDBsum Generate. Sec. struc: 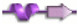 Helices labeled H1, H2, … and strands by their sheets A, B, …; motifs: 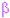 beta turn 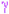 gamma turn 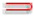 beta hairpin; disulphides: 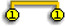 disulphide bond. (**E**,**F**) The 3D structures of *Ss*CTSBa and *Ss*CTSBb were predicted using Phyre2 server. (**G**) Subcellular localization of *Ss*CTSBa and *Ss*CTSBb in HEK293T cells was analyzed by fluorescence microscopy. HEK293T cells were transfected with either GFP-empty and GFP-*Ss*CTSBa or GFP-*Ss*CTSBb. After 48 h, the cells were fixed and the nuclei stained with DAPI. Green fluorescence and blue fluorescence were visualized in the same field (Merge). Scale bars equal 20 μm. (**H**,**I**) Protein–protein interaction analysis of *Ss*CTSBa and *Ss*CTSBb was predicted by STRING 12.0 with the setting of interaction evidence as network edges.

The Pept_C1 domain was present in both *Ss*CTSBa and *Ss*CTSBb as a signal peptide (Figure 1A,B). It could be shown by the secondary structure analysis of *Ss*CTSBa and *Ss*CTSBb that 3 sheets, 3 beta bulges, 3 beta hairpins, 12 strands, and 12 helices were possessed by both of them (Figure 1C,D). Meanwhile, the 3D structure of *Ss*CTSBa was 77% identical to that of c2dcbA, with a confidence of 100%, while that of *Ss*CTSBb was 75% identical to that of c2dcbA, with 100% confidence (Figure 1E,F). Additionally, both *Ss*CTSBa and *Ss*CTSBb might be connected with immune-related signal transductions, which was confirmed by the protein–protein interaction (PPI) network research. As illustrated in Figure 1H, several essential immune-related genes were observed to interact with CTSBa, which were composed of CD74a, mhc2b, mhc2bl, mhc2dab, mhc2dcb, and CTSD. Simultaneously, some of the CTSBb-related genes showed significant roles in host immunity (Figure 1I), including CTSBa, CTSD, CST3, PSAP, and BIDa. In addition, it could be indicated by the subcellular localization assay that *Ss*CTSBa and *Ss*CTSBb were primarily observed within the cytoplasm of HEK293T cells. Moreover, the green fluorescent EGFP protein is primarily localized to the cell nucleus, with some cytoplasmic signal present in the control group (Figure 1G).

### 2.3. Phylogeny and Synteny Analyses

**Figure 2:**
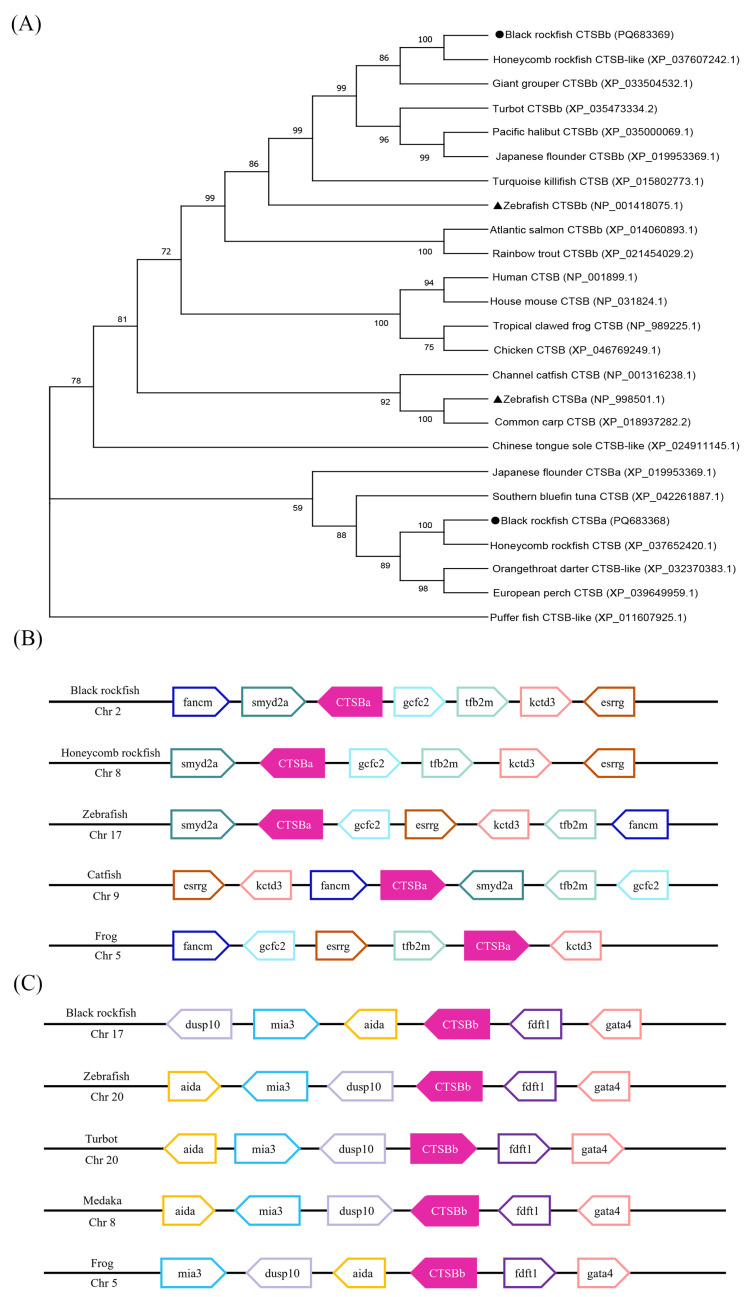
Evolutionary and genomic comparative analysis of *Ss*CTSBa and *Ss*CTSBb. (**A**) Phylogeny analysis of *Ss*CTSBa and *Ss*CTSBb. The phylogenetic tree was constructed based on the amino acid sequences of CTSB from other species using the neighbor-joining method in MEGA X. Gaps were removed by pairwise deletion and the phylogenetic tree was evaluated with 1000 bootstrap replications. The bootstrapping values were indicated by numbers beside the internal branches. *Ss*CTSBa and *Ss*CTSBb were underlined with solid circle. Black solid triangle indicated the zebrafish CTSB genes. (**B**,**C**) Synteny analysis of *Ss*CTSBa and *Ss*CTSBb with other vertebrates. The CTSB genes were highlighted by red color.

The evolutionary connection of CTSB between various species was analyzed in this study, and a phylogenetic analysis was employed to verify the *Ss*CTSBa and *Ss*CTSBb. It was revealed by the results that the CTSB genes can be assigned into two groups. With the support of bootstrapping, the *Ss*CTSBa was firstly grouped with honeycomb rockfish CTSB, followed by southern bluefin tuna CTSB, Japanese flounder CTSBa, puffer fish CTSB-like, and Chinese tongue sole CTSB-like. Subsequently, it was incorporated into the other clade, which was composed of additional teleosts (channel catfish, zebrafish, and common carp) and tetrapods (human, house mouse, tropical clawed frog, and chicken). In contrast, the highest homology was revealed for *Ss*CTSBb in honeycomb rockfish between the teleost-specific CTSBb gene homologues; then, the *Ss*CTSBb was clustered with giant grouper, turquoise killifish, zebrafish, Atlantic salmon, and rainbow trout, before the development of the CTSBa clade (Figure 2A).

A synteny analysis was performed to verify the identification and orthology results of *Ss*CTSBa and *Ss*CTSBb. Firstly, the results of this analysis could offer compelling evidence contributing to the identification of CTSB genes, which could be beneficial to the deep exploration of the evolutionary connection of CTSB. Additionally, five studied vertebrate species exhibited well-conserved synteny with CTSBa and shared the genes *smyd2a*, *gcfc2*, *tfb2m*, *kctd3*, and *esrrg* in their genomic neighborhood, including four teleosts and one tetrapod (Figure 2B). As shown in Figure 2C, CTSBb was conserved among four teleosts and one tetrapod in the analysis of black rockfish, zebrafish, turbot, medaka, and frog with common genes including *aida*, *dusp10*, and *mia3* upstream, and *fdft1* and *gata4* downstream.

### 2.4. Basal Tissue Distribution

**Figure 3:**
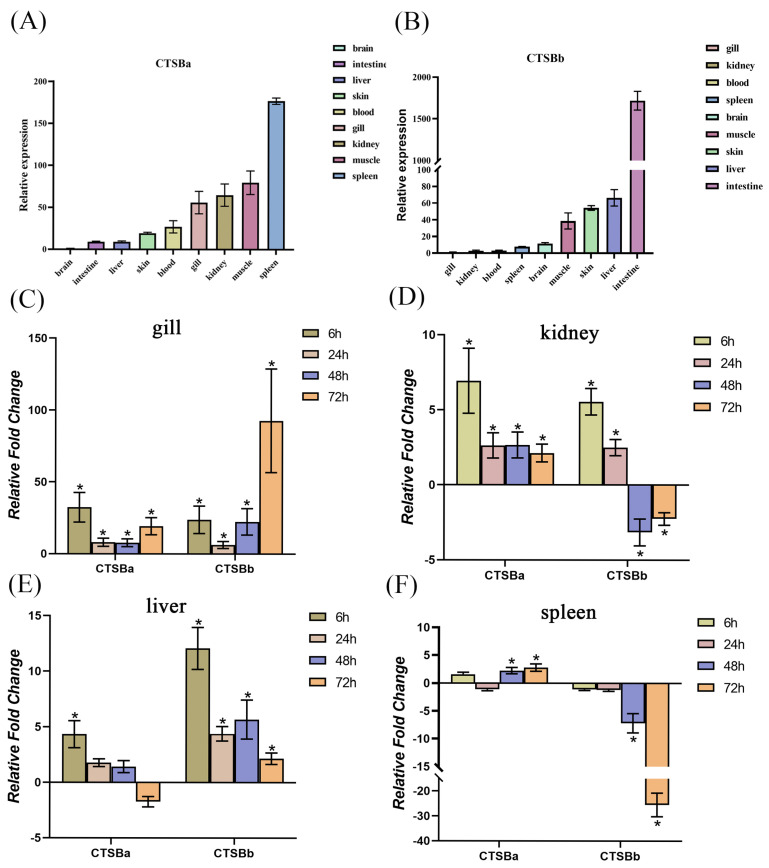
Expression patterns of the *Ss*CTSBa and *Ss*CTSBb in different tissues of healthy black rockfish and their expression levels in gills, kidneys, liver, and spleen at the time points of 6 h, 24 h, 48 h and 72 h following *A. salmonicida* infection. (**A**) The expression levels of *Ss*CTSBa were calibrated against the tissue (brain) that possessed the lowest expression level, and β-actin was used as a reference gene. (**B**) The expression levels of *Ss*CTSBb were calibrated against the tissue (gills) that possessed the lowest expression level, and β-actin was used as the reference. (**C**–**F**) Expression profiles of *Ss*CTSBa and *Ss*CTSBb were measured in gills, kidneys, liver, and spleen at the time points of 6 h, 24 h, 48 h, and 72 h following *A. salmonicida* infection. The fold change was obtained as the expression ratio at a specific time nodes versus the control, and normalized to the expression of the β-actin housekeeping gene. The results are presented as mean ± SD of fold changes and * indicates statistical significance at *p* < 0.05.

qPCR was employed to evaluate the tissue distribution of *Ss*CTSBa and *Ss*CTSBb in all of the tested tissues (kidneys, skin, blood, gills, brain, liver, spleen, intestine, and muscle). Both genes were ubiquitously expressed, with detectable transcript levels in all examined tissues. As shown in Figure 3A, the expression of *Ss*CTSBa in brain showed the lowest level; thus, it was used as the baseline. The highest expression of *Ss*CTSBa could be observed in spleen, followed by muscle, kidneys, gills, blood, skin, liver, and intestine. Notably, the expression of *Ss*CTSBb in the gills showed the lowest level, and it was employed as the baseline. Additionally, the highest expression of *Ss*CTSBb could be observed in the intestine, followed by the liver, skin, muscle, brain, spleen, blood, and kidneys (Figure 3B).

### 2.5. Expression Profiles of SsCTSBa and SsCTSBb Following A. salmonicida

After infection with *A. salmonicida*, qPCR was applied in the analysis to evaluate the expression (Figure 3C–F). Overall, the expression level of *Ss*CTSBa within all tissues showed a significant up-regulated trend with the infection of *A. salmonicida*. In contrast, the expression level of *Ss*CTSBb was up-regulated in gills and liver at all tested time points, while it was down-regulated at 48 and 72 h in kidneys and spleen. In detail, the expression of *Ss*CTSBa in gills was up-regulated at 6 h, 24 h, 48 h, and 72 h, and the peak occurred in 6 h (32.34-fold), as shown in Figure 3C. In contrast, the *Ss*CTSBb showed an up-regulated trend in gills, and the peak occurred at 72 h (92.72-fold). Simultaneously, the expression of *Ss*CTSBa in kidneys and *Ss*CTSBb in liver showed obvious up-regulated trends at each time node (Figure 3D,E). However, although the expression of *Ss*CTSBb firstly up-regulated in the kidneys at 6 and 24 h, it showed an obvious decreasing trend to −3.15-fold at 48 h, and it finally increased to −2.25-fold at 72 h (Figure 3D). In the spleen, the expression level of *Ss*CTSBa was up-regulated at 48 h (2.24-fold) and 72 h (2.78-fold), while that of *Ss*CTSBb was down-regulated at 48 h (−7.15-fold), and sharply dropped at 72 h (−25.47-fold) (Figure 3F). Overall, these findings could further suggest that *Ss*CTSBa and *Ss*CTSBb might be connected with the infection response.

### 2.6. Binding Abilities of rSsCTSBa and rSsCTSBb to Microbial Ligands and Bacteria

**Figure 4:**
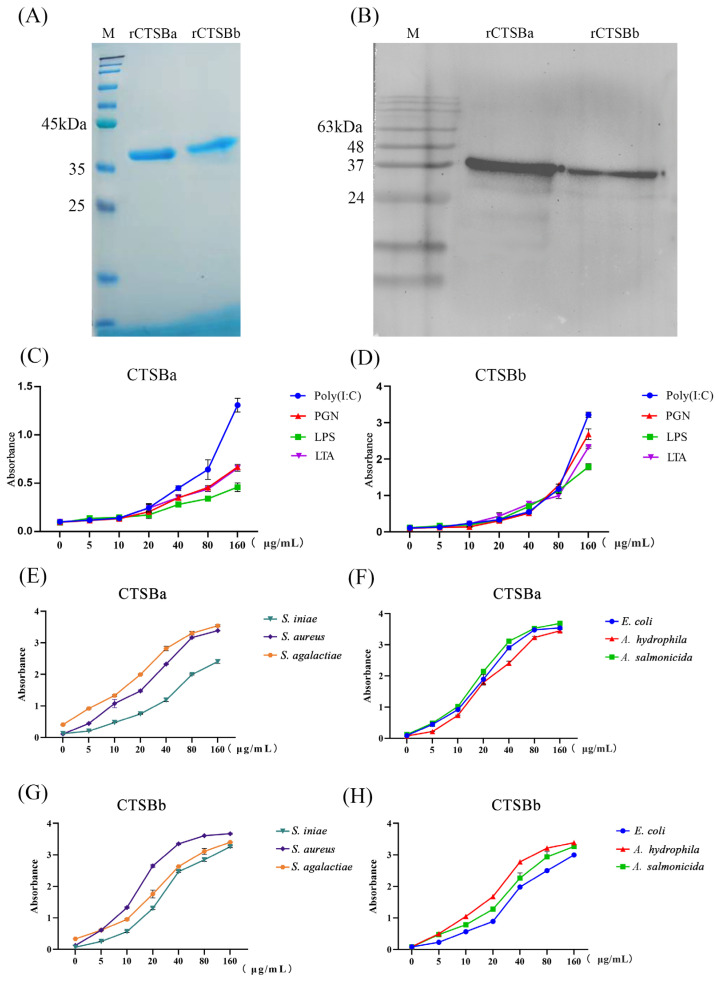
Purification of r*Ss*CTSBa and r*Ss*CTSBb and analysis of their binding abilities with microbial ligands and bacteria. (**A**) SDS-PAGE analysis of r*Ss*CTSBa and r*Ss*CTSBb. The r*Ss*CTSBa and r*Ss*CTSBb analyzed by SDS-PAGE and viewed after staining with Coomassie brilliant blue R-250. (**B**) Western blot analysis of r*Ss*CTSBa and r*Ss*CTSBb. M: Protein marker. (**C**,**D**) Results of the vitro binding assay of r*Ss*CTSBa and r*Ss*CTSBb on microbial ligands, including Poly(I:C), PGN, LPS, and LTA by ELISA. (**E**–**H**) The vitro binding ability of r*Ss*CTSBa and r*Ss*CTSBb with Gram-positive bacteria (*S. aureus*, *S. agalactiae*, and *S. iniae*) or Gram-negative bacteria (*E. coli*, *A. hydrophila*, and *A. salmonicida*) by ELISA.

r*Ss*CTSBa and r*Ss*CTSBb were successfully purified with the IPTG-mediated induction. Additionally, the distinct bands for r*Ss*CTSBa and r*Ss*CTSBb were analyzed by the SDS-PAGE, and each of them showed an approximate molecular weight of 36 kDa (Figure 4A). Notably, the antisera showed a distinct reaction with r*Ss*CTSBa and r*Ss*CTSBb with a molecular weight of around 36 kDa, which was in agreement with the prediction as shown in the WB profiles (Figure 4B). In addition, in vitro assays were employed in the assessment of the binding ability of r*Ss*CTSBa and r*Ss*CTSBb in this study. It could be demonstrated by the absorbance results that r*Ss*CTSBa and r*Ss*CTSBb showed dose-dependent binding with Poly(I:C), LPS, PGN, and LTA, as well as with all test strains of bacteria, including Gram-negative bacteria (*A. salmonicida*, *A. hydrophila*, and *Escherichia coli*) and Gram-positive bacteria (*Streptococcus agalactiae*, *Staphylococcus aureus*, and *Streptococcus iniae*) (Figure 4C–H). Additionally, both r*Ss*CTSBa and r*Ss*CTSBb possessed the highest affinity to Poly(I:C) among the four examined ligands. Furthermore, *Ss*CTSBb showed a higher binding ability to the four ligands compared with that of *Ss*CTSBa (Figure 4C,D). In terms of the binding ability to Gram-positive bacteria, r*Ss*CTSBa showed the strongest combination with *S. agalactiae* followed by *S. aureus* and *S. iniae*, whereas r*Ss*CTSBb had the best binding ability with *S. aureus* followed by *S. agalactiae* and *S. iniae* (Figure 4E,G). Regarding the binding ability to Gram-negative bacteria, r*Ss*CTSBa showed the strongest combination with *A. salmonicida* followed by *E. coli* and *A. hydrophila*, while r*Ss*CTSBb had the best binding ability with *A. hydrophila* followed by *A. salmonicida* and *E. coli* (Figure 4F,H).

### 2.7. Agglutination Assay with Bacteria

**Figure 5:**
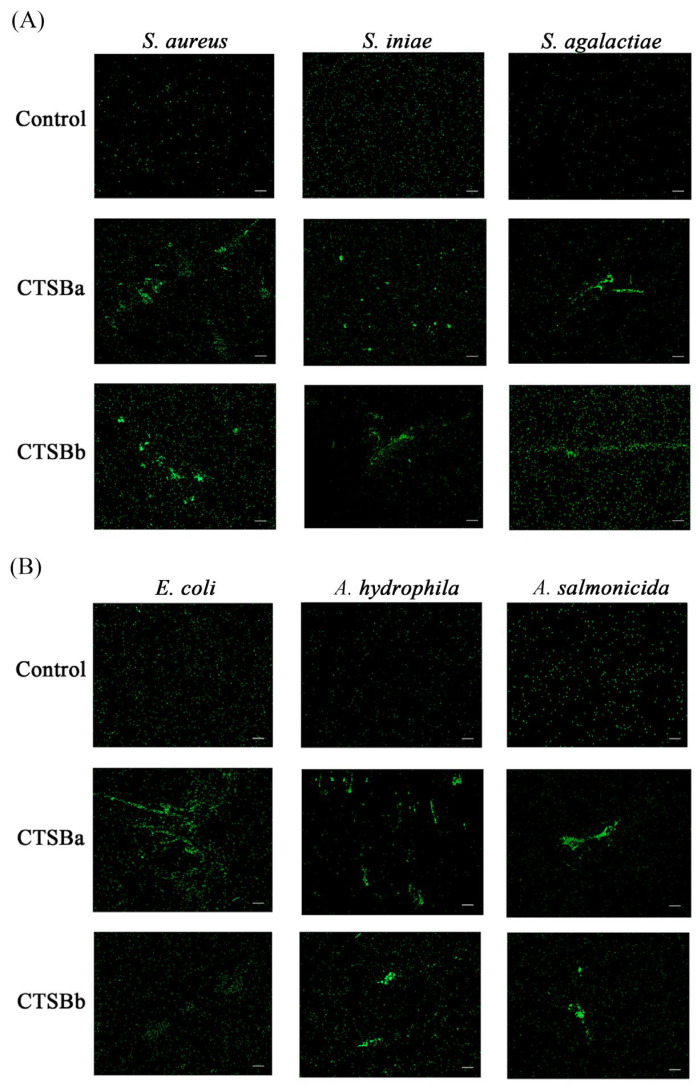
Agglutination assay with bacteria were analyzed by fluorescence microscopy. (**A**) The r*Ss*CTSBa and r*Ss*CTSBb were labeled by FITC and incubated with Gram-positive bacteria (*S. aureus*, *S. iniae* and *S. agalactiae*). (**B**) The r*Ss*CTSBa and r*Ss*CTSBb were labeled by FITC and incubated with Gram-negative bacteria (*E. coli*, *A. hydrophila*, *A. salmonicida*). A volume of 25 μL of mixed FITC dye and 10 μL of bacteria incubated as the control. Scale bars represented 20 μm.

To investigate the coagulant capacity of r*Ss*CTSBa and r*Ss*CTSBb with the bacteria, r*Ss*CTSBa and r*Ss*CTSBb were labeled with FITC and coagulated with Gram-negative bacteria (*A. salmonicida*, *A. hydrophila* and *E. coli*) and Gram-positive bacteria (*S. agalactiae*, *S. aureus*, and *S. iniae*) (Figure 5A,B). Simultaneously, bacteria and FITC dye were added as negative controls; alongside, the observation of green fluorescence was observed by an inverted fluorescence microscope. Additionally, *Ss*CTSBb and Gram-positive/negative bacteria incubation displayed oligomerization (speck structures) except for *E. coli* versus the control groups. In contrast, the Gram-positive bacteria and Gram-negative bacteria were clearly aggregated by *Ss*CTSBa.

## 3. Discussion

Cathepsin B (CTSB) is referred to as a cysteine protease of the cathepsin family, which features a Cys-His double amino acid group within its active center [26]. Additionally, it can serve as both an exopeptidase and endopeptidase [2] and shows crucial functions in various pathological and physiological processes. Under normal physiological conditions, CTSB can facilitate the degradation of the proteins within the lysosome [27]. However, CTSB engages in physiological processes outside the lysosome under pathological conditions [28]. In mammals, CTSB has been connected to cell death by the mechanisms of triggering apoptotic factors upstream of executioner [29] or directly binding and activating caspases [30,31,32]. Additionally, it has been indicated that CTSB could regulate apoptosis in follicles during serum-starvation in *Danio rerio* [33]. In *Apostichopus japonicus*, the down-regulated *Aj*CTSB significantly inhibited coelomocyte apoptosis and influenced the expression of cytochrome c1 and cytochrome c-1/2 genes [34,35]. Due to the involvement in immune-related activities, two CTSB homologues were examined in black rockfish, and the analysis was focused on their immune-related functions and expression profiles in both healthy and bacterially infected fish. Additionally, the abilities to bind and agglutinate microbial ligands and bacteria were characterized in the research. This study could offer a preliminary insight into the immunological participation of CTSB in teleost species, and the results revealed that only one CTSB gene was observed in mammalian species before this study. However, two CTSB homologues can be found in teleosts, including rainbow trout, zebrafish, carp and channel catfish [18,21]. Additionally, CTSBb can only be found in teleosts through lineage-specific tandem gene duplications, which suggests that gene duplication might be the predominant evolutionary mechanism for the adaption of fish to the environment.

The deduced polypeptides of *Ss*CTSBa and *Ss*CTSBb both consisted of 330 amino acids with similar molecular properties (theoretical pI, molecular weight, and instability index) to those in other teleosts [36,37,38]. Moreover, it was revealed by the structural analysis that *Ss*CTSBa and *Ss*CTSBb possessed the typical structural features of the papain family, including a peptidase-C1 domain (Figure 1A,B). Additionally, various active site locations were shown by different species in their amino acid sequences, which were composed of the pacific abalone (*Haliotis discus hannai*), belonging to gastropods [39], and the large yellow croaker [8] and golden pompano [21], belonging to teleosts. *Ss*CTSBa possessed three active site residues and two N-glycosylation sites (Table 1). Nevertheless, three active site residues and only one N-glycosylation site were observed in *Ss*CTSBb (Table 1). Moreover, the activity of cysteine-like proteases with conserved histidine, cysteine, and asparagine residues could significantly affect the stability of enzyme catalytic sites. It was revealed by Chen et al. [16] that His277 was essential for enzyme activity due to its ability to form an imidazole ring with Asn. Additionally, the N-glycosylation site showed great importance in the development of the organism without relation to the enzyme activity [40,41]. Cathepsins are crucial in the process of lysosomal transport and immune response to pathogen infections [42]. In addition, the duplication of CTSB in black rockfish parallelled the gene duplication that was observed in mammal-specific CC chemokines following the divergence of mammalian and bird ancestral lineages [43,44]. The parallel gene duplication events observed for both CTSB and CC chemokine genes in these distinct lineages likely reflect convergent evolutionary pressures for immune system complexity. Following gene duplication in teleost fish, CTSB may have undergone functional diversification similar to that observed in mammalian CC chemokines.

It could be indicated by the phylogenetic analysis (Figure 2A) and syntenic patterns (Figure 2B,C) of the selected species that the orthology and synteny relationship of the CTSB genes are conserved. Overall, the identification of the CTSB genes of black rockfish could be confirmed by the combined molecular characteristics, numerous species comparisons, phylogenetic analysis, and syntenic analysis, which also showed strong homology with the counterparts in other species. Additionally, the conservation of the structural characteristics of *Ss*CTSBa and *Ss*CTSBb could indicate potential functional similarities with other species.

The interacting genes of *Ss*CTSBa and *Ss*CTSBb were predicted by the PPI network analysis for the deep exploration of their functions in immune-related signaling pathways. The interacting genes of *Ss*CTSBa are composed of CD74a, mhc2b, mhc2bl, mhc2dab, mhc2dcb, and CTSD, while the interacting genes of *Ss*CTSBb include CTSBa, CTSD, CST3, PSAP, and BIDa (Figure 1H,I), among which the four MHC class II molecules (mhc2b, mhc2bl, mhc2dab, and mhc2dcb) are expressed by APCs and exhibit antigen presentation characteristics [45,46,47]. CD74a is referred to as the key bioactive molecule, and it was crucial for the assembly and trafficking of MHC II molecules during antigen presentation [48]. Notably, CTSB/D/E/F/K/L/S have been reported to be connected with the degradation of associated chaperones (invariant chain[li]) and antigen processing in mammals, as demonstrated by vitro studies [3]. Therefore, it was assumed that *Ss*CTSBa and *Ss*CTSBb might be involved in the fish MHC II antigen presentation pathway with CTSD and CD74a, and the precise mechanism of their interaction should be explored. Different from CTSB, which is referred to as a cysteine protease, CTSD is an aspartate protease [49]. It has been proven that PSAP precursors can be divided by CTSD into four small saposins based on PSAP localization [50]. In addition, the lysosomal function and activity of CTSB and CTSD might be associated with saposin-C [51]. It has been revealed by previous studies that autophagic flux and cholesterol metabolism are dramatically impacted by defective endolysosomes with reduced amounts of mature CTSB and CTSD [52]. CST3 was considered to be a cysteine protease inhibitor that controlled lysosomal enzyme activity, and it was confirmed to be linked with neurodegeneration. In vitro, inhibitory effects of CST3 on proteinase are weakened when it is bound to PSAP [53]. Notably, a PPI analysis was conducted according to the *D. rerio* and *C. semilaevis* datasets due to the limited identification of black rockfish protein data, and the PPI databases of black rockfish and other fish species need to be established further. On account of the report that CTSB in cytoplasm could result in cell death [54], a chimeric GFP protein was constructed to examine the location of *Ss*CTSBa and *Ss*CTSBb in cells. According to the findings, it was revealed by green fluorescence that *Ss*CTSBa and *Ss*CTSBb were observed in the cytoplasm, and the *Ss*CTSBa and *Ss*CTSBb in the cytoplasm might participate in transportation or degradation processes, which corresponded to the PPI results. It has been demonstrated that the proteolytic enzyme CTSB was essential to antigen processing by breaking down both antigens and B cell receptors (BCRs), which could make it easier for Human Leukocyte Antigen (HLA) class II molecules to present peptide fragments [55].

Ubiquitous expression signatures of *Ss*CTSBa and *Ss*CTSBb were observed, and the widespread expression of CTSB in various tissues was in agreement with previous research [8,41,56,57,58]. Similarly to CTSB in Nile tilapia [41], the expression level of *Ss*CTSBa follows this order: spleen, muscle, kidneys, gills, blood, skin, liver, intestine, and brain (Figure 3A). In contrast, the intestine showed the highest expression of *Ss*CTSBb, which was followed by the liver, skin, muscle, and brain (Figure 3B). Additionally, the gills showed the highest CTSB expression out of the other tissues examined in orange-spotted groupers, followed by the head, kidneys, spleen, and heart [56]. Notably, their potential functions are suggested by the relatively high expression levels in immune-related tissues including the spleen, intestine, muscle, liver, kidneys, skin, and gills. In addition, the spleen is considered to be an important immune organ in fish, and shows significant roles in the immune response. The highest expression level of CTSB in the spleen could be attributed to its involvement in immune processes. In recent years, CTSB and other cathepsins have been discovered to show important effects in both intracellular and extracellular matrix (ECM) degradation [59]. Additionally, the intestine is considered the primary site for digestion and absorption in fish, and it might involve CTSB during the process of food digestion and nutrient absorption within the intestinal tract. In addition, it might be beneficial to the breaking down of protein components in food, contributing to access for absorption by intestinal cells. CTSB was found to be connected with proteins that digest yolk, which benefits the quality and viability of eggs, and also provides an energy source for embryos of fish species [60,61].

It was reported that CTSB might induce the host immune response stimulated by microbial ligands or pathogens [14,16,18,62]. For instance, CTSB could promote TNF-α post-translational processing and contribute to its synthesis in response to LPS stimulation, which results in the host’s immunological response to infections [62]. The expression of CTSBa in channel catfish mucosal tissues was induced following *Edwardsiella ictaluri* and *Flavobacterium columnare* challenge [18]. In *C. semilaevis*, the expression of CTSB in the head kidney was up-regulated to 25-fold at 4 h after *Vibrio anguillarum* challenge [16]. In *Oplegnathus fasciatus*, the expression level of CTSB in the spleen was up-regulated by 2.7-fold at 24 h after *E. tarda* infection [14]. Corresponding to the findings above, the expression of *Ss*CTSBa in our current investigation showed an up-regulated trend after *A. salmonicida* infection, particularly in the gills of all of the examined tissues at the majority of the time points (Figure 3C). Nevertheless, the expression level of *Ss*CTSBb showed an obvious down-regulation trend in the spleen, while it exhibited up-regulation in the gills and liver (Figure 3C,E,F). It has been revealed by previous research that the overexpression of CTSB could improve the autophagic flux in endothelial cells and reduce cell apoptosis under hypoxic conditions [63]. Although this research mainly focused on hypoxic environments, similar mechanisms could be found in certain bacterial infections. A deficiency of CTSB was confirmed to inhibit intracellular infection with *Brucella*. With the development of infection, *A. salmonicida* might inhibit the autophagy process in host cells to down-regulate the expression of *Ss*CTSBb, thereby avoiding the surveillance of the host immune system. Additionally, Ectromelia virus has been shown to inhibit the expression of CTSB in murine conventional dendritic cells, thereby facilitating its replication process [64]. However, in *Mycobacterium tuberculosis*-infected rabbits, increased CTSB gene expression was observed in the lungs, and increased protein levels were observed in plasma from patients with active tuberculosis [65]. Thus, the changes in the expression levels of CTSB might vary according to the type of pathogen and host response, and it was relatively common for the expression level of cathepsin genes to be induced after bacterial infection. In addition, such general up-regulated expression levels could also be observed for CTSK and CTSS in black rockfish following *V. anguillarum* infection [38,66]. Notably, the expression of CTSK in the spleen of black rockfish showed an upward trend and peaked at 24 hpi (24.8-fold) [66], while the expression of CTSS in the spleen was also obviously up-regulated, with the maximum induction at 12 hpi (26.5-fold) [38]. The expression levels in the four primary immune-related tissues (gills, kidneys, liver, and spleen) induced by pathogen infection appeared to be different, which indicated the participation of CTSB in the immunological response.

Regarding the deep exploration of the immunological participation of *Ss*CTSBa and *Ss*CTSBb in the host defense against different pathogens, r*Ss*CTSBa and r*Ss*CTSBb were purified, alongside the application of four representative microbial ligands in an in vitro binding experiment. According to the results, both r*Ss*CTSBa and r*Ss*CTSBb were efficiently expressed in BL21 (DE3), and exhibited high binding ability with all the ligands, particularly Poly(I:C) (Figure 4C,D). Additionally, Poly(I:C) is an interferon inducer featuring broad-spectrum antiviral and immunological regulatory properties. And it could indicate the significant participation of *Ss*CTSBa and *Ss*CTSBb in the immune response. Poly(I:C)-induced microglia activation, which might release proinflammatory factors, showed a decreasing trend in BV2 cells with the inhibition of CTSB activity by inhibitor CA-074 after 24 h, and the initial phases of activation-induced microglia death could indicate active functions for cathepsin X [67]. Regarding the flounder embryonic cells, CTSB could be induced by Poly(I:C), LPS, and viruses in vitro and in vivo, and Poly(I:C) is considered to be one of the most effective inducers during the transcription of CTSB [20]. In C2C12 myotubes, CTSB could promote the process of TNF-α post-translation and contribute to its synthesis in response to LPS stimulation, which resulted in a host immunological response to infections [68]. In addition, both r*Ss*CTSBa and r*Ss*CTSBb exhibited strong agglutination effects on *S. aureus*, *S. iniae*, *S. agalactiae*, *A. hydrophila*, and *A. salmonicida* (Figure 5A,B). The results above were consistent with their binding abilities to all of the examined bacteria (Figure 4E–H). Additionally, CTSB was confirmed to degrade various extracellular matrix components in vitro according to existing studies, which were composed of glycosaminoglycans, elastin, and collagen. In rheumatoid arthritis and osteoarthritis, CTSB was found to contribute to joint damage by degrading type II collagen and proteoglycans in articular cartilage [59]. Previously, the CTSK and CTSS of black rockfish were observed to agglutinate the bacteria (*E. coli*, *V. anguillarum*, and *S. aureus*) and bound with LPS and PGN [38,66]. In addition, CTSZ showed a high affinity for LPS, PGN, and LTA, and it demonstrated an effective coagulant capacity for Gram-negative and Gram-positive bacteria in turbot [69]. The differences between CTSB and CTSK/CTSS/CTSZ indicated their important immunological participation in the host defense system. Moreover, the black rockfish that were treated with CTSS knockdown showed clearly higher bacteria amounts versus the control after the infection [38], while the fish that underwent the CTSK knockdown exhibited significantly lower bacterial amounts than the control group [66]. Overall, the opposing roles of various cathepsins in innate immunity could be demonstrated by these findings, which could indicate that *Ss*CTSBa and *Ss*CTSBb might further serve as targets within the innate immune response.

## 4. Materials and Methods

### 4.1. Sequence Identification

CTSB gene sequences from various vertebrate species were used as queries to search against the black rockfish genome database based on the BLAST program version 2.16 [70,71]. The transcriptome databases were developed in our laboratory and utilized in this study. Subsequently, duplicates were removed from the initial retrieved sequence pool that was recovered by Clustal Omega (https://www.ebi.ac.uk/jdispatcher/msa/clustalo, accessed on 10 August 2024), ensuring the identification of each sequence as one based on its genomic location. Additionally, the sequences mentioned above were initially deduced with the support of the ORF finder application (https://www.ncbi.nlm.nih.gov/orffinder/, accessed on 16 August 2024) and subsequently verified through BLASTP against the NCBI database. In addition, SMART (http://smart.embl-heidelberg.de/, accessed on 24 August 2024) was employed in the operation of the conserved domains and signal peptides.

### 4.2. Sequence Analyses

A range of bioinformatic analyses were analyzed for the characteristics of CTSB. Additionally, the ExPASy [72] and PROSITE server were used to capture the physiochemical properties and characteristic signatures of CTSB. Generally, the proteins were considered to be stable with an instability index less than 40. In addition, the aliphatic index was determined by the relative volume of aliphatic side chains, and it showed crucial effects in the enhancement of the thermostability of globular proteins. The GRAVY score was calculated by averaging the total hydropathy values of amino acids. The ideational 3D protein structure was generated by the Phyre2 server [73]. Additionally, the protein–protein interaction was analyzed by performing a BLAST search of the amino acid sequences of *Ss*CTSBa and *Ss*CTSBb against *Danio rerio* or *Cynoglossus semilaevis* based on STRING software 12.0.

### 4.3. Subcellular Localization

The HEK293T cell line was obtained from the American Type Culture Collection (ATCC, CRL-3216, RRID:CVCL_0063), located in Manassas, Virginia, USA. HEK293T cells were maintained in Dulbecco’s Modified Eagle Medium (DMEM, Gibco, Grand Island, NY, USA) supplemented with 10% fetal bovine serum (FBS, Gibco) and 1% penicillin/streptomycin (Gibco). The pEGFP-N2-*Ss*CTSBa/b constructs were generated by cloning the full-length *Ss*CTSBa and *Ss*CTSBb coding sequences (amplified from black rockfish cDNA) into the BamHI and Nhel sites of the pEGFP-N2 vector (Clontech, Shiga Prefecture, Kyoto, Japan). The constructs were verified by Sanger sequencing. HEK293T cells were seeded on sterile coverslips in the 24-well plates at a density of 1 × 10^5^ cells per well to locate *Ss*CTSBa and *Ss*CTSBb. After 24 h, cells were transfected with 1 μg of either the pEGFP-N2-empty or pEGFP-N2-*Ss*CTSBa/b plasmid utilizing TurboFect™ transfection reagent. At 48 h post-transfection, the cells were put on slides with Fluoroshield consisting of DAPI (Sigma-Aldrich, Darmstadt, Germany), and examined with an inverted fluorescence microscope (objective 40×). The localization pattern was demonstrated through three independent experiments, confirming the reliability of our conclusion. For each experiment, at least 30 successfully transfected cells (with fluorescence intensity significantly higher than background) were analyzed, with approximately 80% fluorescence-positive cells determined by manual inspection. The pEGFP-N2-empty vector is commercially available (Clontech, Shiga Prefecture, Japan). All custom plasmids (pEGFP-N2-*Ss*CTSBa/b) will be made freely available upon request to the corresponding author.

### 4.4. Phylogeny and Synteny Analyses

A phylogenetic tree was generated with the application of amino sequences of CTSB in black rockfish and other various species, and the sequences were assessed including human (*Homo sapiens*), house mouse (*Mus musculus*), tropical clawed frog (*Xenopus tropicalis*), chicken (*Gallus gallus*), honeycomb rockfish, orangethroat darter (*Etheostoma spectabile*), European perch (*Perca fluviatilis*), Southern bluefin tuna (*Thunnus maccoyii*), Japanese flounder (*Paralichthys olivaceus*), puffer fish (*Takifugu rubripes*), Chinese tongue sole (*Cynoglossus semilaevis*), channel catfish, zebrafish, common carp (*Cyprinus carpio*), Atlantic salmon (*Salmo salar*), rainbow trout (*Oncorhynchus mykiss*), turquoise killifish (*Nothobranchius furzeri*), giant grouper (*Epinephelus lanceolatus*), turbot (*Scophthalmus maximus*), and Pacific halibut (*Hippoglossus stenolepis*). Additionally, the protein sequences were aligned using the ClustalW2 program [74]. Phylogenetic analysis was conducted in MEGA X software to examine the evolutionary connection with the counterpart molecules based on the neighbor-joining method with 1000 bootstrapping replications [75]. Poisson distance was employed for the correction of the analysis, and the gaps were addressed via pairwise deletion.

Syntenic analysis was operated for the orthology analysis of the two CTSB genes in black rockfish, which was based on the comparison between the neighboring genes of *Ss*CTSBa and *Ss*CTSBb and those of zebrafish, frog, catfish, turbot, medaka, and honeycomb rockfish in the FGENESH program [76]. Additionally, the BLASTP was used to annotate the identified protein sequences according to the NCBI database, and the Genomicus and Ensembl database were employed in the evaluation of the conserved syntenic pattern of CTSB genes [77].

### 4.5. Sample Collection of Healthy Black Rockfish

Samples from nine tissues of healthy black rockfish were obtained to describe the expression profiles of *Ss*CTSBa and *Ss*CTSBb, and the black rockfish were purchased from a black rockfish hatchery in Haiyang (Shandong Province, China), in which the fingerlings showed an average weight of 14.8 g and length of 5.6. The fish were acclimated for at least two weeks in a recirculating seawater system before the collection of tissues. The tissue samples were obtained from 30 healthy black rockfish that had first been anesthetized with seawater containing 100 mg/L of tricaine methane sulfonate (MS-222). All samples were quickly frozen in liquid nitrogen and stored at −80 °C until the RNA preparation.

### 4.6. Bacterial Challenge and Sample Collection

The bacterium *A. salmonicida* was selected to perform the bath challenge for the expression of *Ss*CTSBa and *Ss*CTSBb. Additionally, the bacteria were biochemically verified and re-isolated from fish exhibiting symptoms following a pre-challenge before the cultivation. The infection of symptomatic fish with *A. salmonicida* was checked during the challenge. Additionally, the fish were bathed in the bacterial solution with the concentration of 5 × 10^6^ CFU/mL for 2 h during the experiment, and the gills, kidneys, liver, and spleen tissues were obtained from 15 fish (5 fish per replicate) at intervals of 6 h, 24 h, 48 h, and 72 h after treatment with 100 mg/L MS-222. Samples were promptly frozen by liquid nitrogen and maintained at −80 °C until the RNA preparation.

### 4.7. Total RNA Extraction and Real-Time PCR Analyses

Trizol^®^ Reagent (Invitrogen, Carlsbad, CA, USA) was employed in the extraction of total RNA by the provided procedure, and 1% agarose gels were used to track RNA contamination and degradation. The concentration value was assessed using the DS-11 Spectrophotometer (Denovix, Wilmington, DE, USA), and the A260/280 ratios for all extracted samples were above 1.8. Then, the expression patterns were determined by qPCR methods. Primer 6 online program was employed in the design of specific primers for the CTSB gene sequences of black rockfish, and the β-actin gene was used as the reference for normalizing the expression levels (Table 2). The synthesis of cDNA was carried out using the PrimeScript RT reagent Kit (TaKaRa, Dalian, China) following the manufacturer’s instructions. Additionally, qPCR was completed using the CFX96 detection system (BioRad Laboratories, Hercules, CA, USA) based on the SYBR ExScript qPCR Kit (Takara, Dalian, China), as instructed. Standard curves were developed in this study to determine the amplification efficiency, threshold values, and the formula for the calculation of relative gene copy numbers. Then, the relative mRNA expression was analyzed in triplicate. All the values were presented as means ± SD, and fold differences were determined with the aid of the Relative Expression Software Tool (REST) to ensure statistical significance (*p* < 0.05) [78].

### 4.8. Purification of Recombinant SsCTSBa and SsCTSBb Protein (rSsCTSBa and rSsCTSBb)

The DNA fragment encoding *Ss*CTSBa and *Ss*CTSBb was amplified by the high-fidelity DNA polymerase (TaKaRa, Dalian, China) and specific primers (*Ss*CTSBa-Pr F/R and *Ss*CTSBb-Pr F/R, Table 2). The target PCR product was subcloned into the PET32a-modified HTH expression vector after the sequencing process. Then, *E. coli* BL21 (DE3) was transformed by the resulting HTH-*Ss*CTSBa and HTH-*Ss*CTSBb plasmids, which expressed histidine-tagged r*Ss*CTSBa and r*Ss*CTSBb. Subsequently, the cells were fostered in the LB medium and induced by 0.5 mM IPTG. Next, the recombinant *Ss*CTSBa and *Ss*CTSBb proteins (r*Ss*CTSBa and r*Ss*CTSBb) were purified with the application of nickel-nitrilotriacetic acid columns (GE Healthcare, Piscataway, NJ, USA). In addition, the screening samples were evaluated by 12% SDS-PAGE. The concentration of the fusion proteins was tested using Bradford’s technique. Moreover, the existence of the purified r*Ss*CTSBa and r*Ss*CTSBb was confirmed by the Western blot analysis. The HTH-*Ss*CTSBa/b constructs were generated by cloning the full-length *Ss*CTSBa and *Ss*CTSBb coding sequences (amplified from black rockfish cDNA) into the BamHI and KpnI sites of the HTH vector. All custom plasmids (HTH-*Ss*CTSBa/b) will be provided upon request to the corresponding author.

### 4.9. Binding Abilities of rSsCTSBa and rSsCTSBb with Microbial Ligands and Bacteria

Each well of the 96-well microtiter plates (Corning, NY, USA) was covered by 0.25 μg of lipopolysaccharide (LPS), peptidoglycan (PGN), lipoteichoic acid (LTA), and polyinosinic acid–polycytidylic acid (Poly(I:C)) at 4 °C overnight, as well as Gram-negative bacteria (*E. coli*, *A. hydrophila*, and *A. salmonicida*) and Gram-positive bacteria (*S. aureus*, *S. agalactiae*, and *S. iniae*), respectively. Notably, the plates were washed three times by PBST (0.05% Tween-20 in PBS) and then blocked with 5% BSA at 37 °C for 1 h. Subsequently, 100 μL of the control protein alongside the empty HTH or the purified r*Ss*CTSBa and r*Ss*CTSBb with increased concentration was incorporated into the ligand-coated wells, and incubated at 37 °C for 1 h with four replicates for each concentration, followed by five rounds of washing with PBST. Notably, the concentration levels were designed with the values of 5, 10, 20, and 40 μg/mL in the above experiments. The plate was then incubated at 37 °C for 1.5 h with 100 μL mouse anti-His antibody (Affinity Biosciences, Taizhou, China). After washing three times with PBST, horseradish peroxidase-conjugated goat anti-mouse IgG (Affinity Biosciences, Taizhou, China) was incorporated into the plates. An ELISA plate reader was employed in the measurement of OD_450 nm_ after the termination of reactions with 0.5 M sulfate.

### 4.10. Agglutination of FITC-Labeled rSsCTSBa and rSsCTSBb with Bacteria

Depending on the labeling of r*Ss*CTSBa and r*Ss*CTSBb with fluorescein isothiocyanate (FITC), the agglutination capabilities were investigated in this study. Additionally, *S. aureus*, *S. iniae*, *S. agalactiae*, *E. coli*, *A. hydrophila*, and *A. salmonicida* were cultivated with LB broth in a shaker incubator under the conditions of 180 rpm and 28 °C overnight. After the centrifugation of 5000× *g* for 10 min, the bacteria were resuspended twice in TBS-Ca and adjusted to a concentration of 2.5 × 10^9^ CFU/mL. In addition, the obtained r*Ss*CTSBa and r*Ss*CTSBb were dialyzed against PBS at 4 °C for 12 h, with the adjustment of concentration to 2 mg/mL. The HOOKTM Dye Labelling Kit (G-Biosciences, St. Louis, MO, USA) was then used to label the r*Ss*CTSBa and r*Ss*CTSBb based on the guidelines. Subsequently, the protein solution was mixed with the computed volume of the newly made Dye Labelling Agent solution. After a quick centrifugation and vortex, the sample was finally obtained from the bottom of the tube, and aluminum foil was wrapped around the tube to block the light. The FITC-labeled r*Ss*CTSBa and r*Ss*CTSBb protein solution were centrifuged at 1000× *g* for 6 min by SpinOUTTM columns after one hour of incubation under room temperature conditions to remove the unconjugated dye. Following a one-hour incubation period at room temperature, 10 μL of bacteria was combined with 25 μL of FITC-labeled r*Ss*CTSBa and r*Ss*CTSBb. Microbial suspensions (20 μL) were placed on slides and analyzed using an inverted fluorescence microscope. Additionally, the same treatment was conducted on the negative control, and only 10 μL of bacteria and 25 μL of mixed FITC dye were used in the treatment. The agglutination assay was replicated three times with biological replicates, demonstrating consistent and reliable results. For each bacterial agglutination assay, 10 independent microscopic fields per sample were analyzed. Above 90% of the fields showed comparable agglutination patterns across replicates.

### 4.11. Statistical Analyses

All of the statistical analyses were conducted using SPSS 26.0 software, and a one-way analysis of variance (ANOVA) was employed in the assessments of the study, with *p* < 0.05 representing statistical significance.

## 5. Conclusions

Two cysteine cathepsins from black rockfish, *Ss*CTSBa and *Ss*CTSBb, were thoroughly analyzed in this study. The annotation of the *Ss*CTSB genes was performed in this study based on the sequence structure, phylogenetic, and syntenic analysis, which could effectively verify the conservation of these genes among their counterparts in other tetrapod and teleost species. Additionally, *Ss*CTSBa and *Ss*CTSBb were commonly expressed in all of the tissues, and the highest expression was observed in the spleen and intestine, respectively. Furthermore, the significant variation in the expression levels between the immune-related tissues (kidneys, liver, spleen, and gills) after bacterial infection suggests that these tissues are actively involved in immunological responses. Moreover, CTSB genes in black rockfish showed the ability to bind with microbial ligands or bacteria and diverse agglutination effects on Gram-negative/positive bacteria. Overall, this research could provide evidence for the immunological roles of CTSB, and further research is required for the deep exploration of its functions in the immunity defense system. 

## Figures and Tables

**Table 1:** Primary structural analysis. Properties of *Ss*CTSBa and *Ss*CTSBb were determined by ProtParam.

Analyses	CTSBa	CTSBb
mRNA	993 bp	993 bp
No. of amino acids	330 aa	330 aa
Molecular weight	36.06 kDa	36.06 kDa
Theoretical pI	5.38	5.20
Formula	C_1599_H_2412_N_424_O_486_S_22_	C_1590_H_2411_N_433_O_487_S_21_
Instability index	32.27	31.46
Aliphatic index	69.42	70.94
Grand average of hydropathicity (GRAVY)	−0.260	−0.309
Cysteine proteases active site	3	3
Protein kinase C phosphorylation site	4	4
Casein kinase II phosphorylation site	4	2
N-glycosylation site	2	1
Total number of negatively charged residues (Asp + Glu)	37	37
Total number of positively charged residues (Arg + Lys)	27	24

**Table 2:** Primers used in this study.

Primer	Sequence (5′-3′)
CDS clone	
*Ss*CTSBa-ORF F	CAGTCATTCTCTGTTCTCTGATTCC
*Ss*CTSBa-ORF R	ACACTCGGCAGGAAATCGTATAAAT
*Ss*CTSBb-ORF F	ATTTTGACCAGGACAGACACGAT
*Ss*CTSBb-ORF R	GCAGATGTAAGATTTATGTGGCAAG
qPCR	
*Ss*CTSBa F	CACTCCCAGCTACAAAGTAGAC
*Ss*CTSBa R	CTACTGGGCCGTTCTTGTATAG
*Ss*CTSBb F	TGGGCTGTTATGGTGGTTATC
*Ss*CTSBb R	AGCCGACATTGGAGTTATACAG
β-actin F	GTGCGTGACATCAAGGAGAAGC
β-actin R	TGTTGTAGGTGGTCTCGTGGA
Subcellular localization	
EGFP-*Ss*CTSBa F	CGTCAGATCCGCTAGCATGTGGCGTGCAGCTTTCCT
EGFP-*Ss*CTSBa R	ACGGCCGGTGGATCCGTTTGGGAATCCCCGCC
EGFP-*Ss*CTSBb F	CGTCAGATCCGCTAGCATGCATCCTCTGGCTCTCGTTT
EGFP-*Ss*CTSBb R	ACGGCCGGTGGATCCGGTTGAGTGGGATTCCTGCC
Prokaryotic expression	
*Ss*CTSBa-Pr F	GGTAAAATCGAAGAAGGATCCAGACCCCGCCTCCCACCACTGT
*Ss*CTSBa-Pr R	AGAACCGTTACCAGAGGTACCTTTGGGAATCCCCGCCACAATC
*Ss*CTSBb-Pr F	GGTAAAATCGAAGAAGGATCCCGGCCTAACCTCCCTCATGCCT
*Ss*CTSBb-Pr R	AGAACCGTTACCAGAGGTACCGTTGAGTGGGATTCCTGCCACC

## Data Availability

The original contributions presented in this study are included in the article/Supplementary Materials. Further inquiries can be directed to the corresponding author(s).

## Supplementary Materials

The following supporting information can be downloaded at https://www.mdpi.com/article/10.3390/md23050213/s1. Figure S1: Alignment of the deduced amino acid sequences of *Ss*CTSBa and *Ss*CTSBb with other species. Table S1: Abbreviations and accession numbers of gene names used in phylogenetic tree. Table S2: Abbreviations of gene names used in synteny analysis. Table S3: Abbreviations and accession numbers of gene names used in PPI. Table S4: Abbreviations of gene names used in PPI. Table S5: Abbreviations of gene names and accession numbers used for sequence identification.
