# Supplementary material for: Identification, Expression Profiling, Microbial Binding, and Agglutination Analyses of Two Cathepsin B Genes in Black Rockfish (Sebastes schlegelii)
Source: Mar Drugs. 2025 May 18;23(5):213. doi: 10.3390/md23050213 (PMC12112843; doi:10.3390/md23050213)
Supplement: Supplementary file 1 [file marinedrugs-23-00213-s001.zip › marinedrugs-3577777-supplementary.pdf]

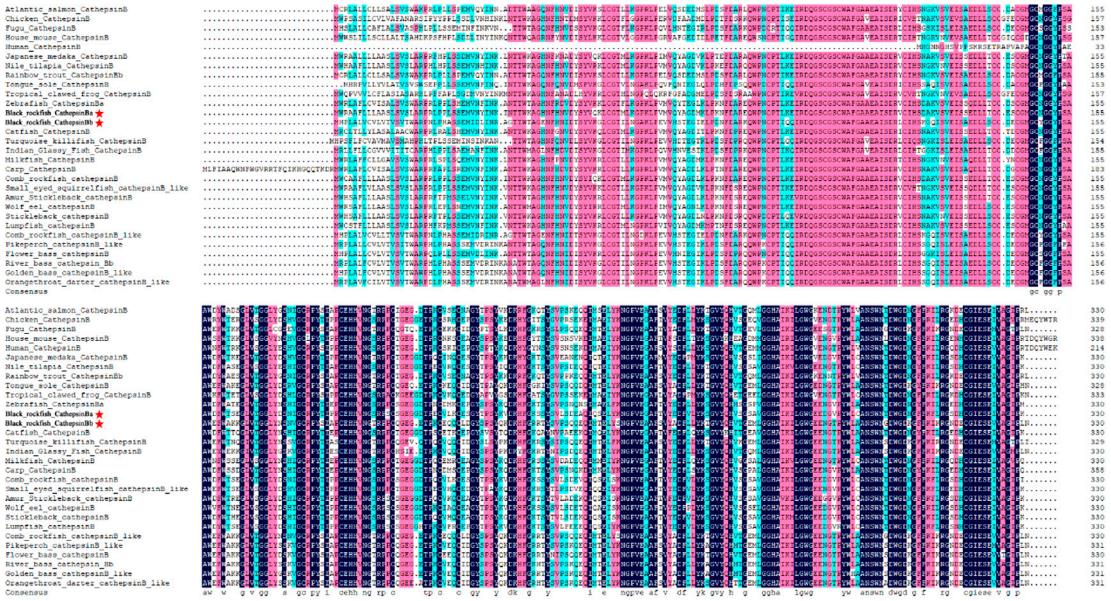

**Figure S1.** Alignment of the deduced amino acid sequences of *Ss*CTSBa and *Ss*CTSBb with other species. Dash denotes gaps introduced for maximum matching. The consensus residues are in black, the residues that are ≥75% identical among the aligned sequences are in pink.

**Table S1. Abbreviations and accession numbers of gene names used in phylogenetic tree.**

| | |
|---|---|
| CTSBa_Black rockfish | PQ683368 |
| CTSBb_Black rockfish | PQ683369 |
| CTSBa_Zebrafish | NP_998501.1 |
| CTSBb_Zebrafish | NP_001418075.1 |
| CTSB_Honeycomb rockfish | XP_037652420.1 |
| CTSB-like_Honeycomb rockfish | XP_037607242.1 |
| CTSB_Common carp | XP_018937282.2 |
| CTSB-like_Orangethroat darter | XP_032370383.1 |
| CTSB_European perch | XP_039649959.1 |
| CTSB_Southern bluefin tuna | XP_042261887.1 |
| CTSBa_Japanese flounder | XP_019935873.1 |
| CTSBb_Japanese flounder | XP_019953369.2 |
| CTSB-like_Puffer fish | XP_011607925.1 |
| CTSB-like_Chinese tongue sole | XP_024911145.1 |
| CTSB_Channel catfish | NP_001316238.1 |
| CTSB_Human | NP_001899.1 |
| CTSB_House mouse | NP_031824.1 |
| CTSB_Tropical clawed frog | NP_989225.1 |
| CTSB_Chicken | XP_046769249.1 |
| CTSBb_Atlantic salmon | XP_014060893.1 |
| CTSBb_Rainbow trout | XP_021454029.2 |
| CTSB_Turquoise killifish | XP_015802773.1 |
| CTSBb_Giant grouper | XP_033504532.1 |
| CTSBb_Turbot | XP_035473334.2 |
| CTSBb_Pacific halibut CTSBb | XP_035000069.1 |

**Table S2. Abbreviations of gene names used in synteny analysis.**

| Gene abbreviation | Full name |
|---|---|
| FANCM | FA complementation group M |
| smyd2a | SET and MYND domain containing 2a |
| gcfc2 | GC-rich sequence DNA-binding factor 2 |
| tfb2m | transcription factor B2, mitochondrial |
| KCTD3 | potassium channel tetramerization domain containing 3 |
| esrrg | estrogen related receptor gamma |
| dusp10 | dual specificity phosphatase 10 |
| mia3 | MIA SH3 domain ER export factor 3 |
| aida | axin interactor, dorsalization associated |
| fdft1 | farnesyl-diphosphate farnesyltransferase 1 |
| gata4 | GATA binding protein 4 |

**Table S3. Abbreviations and accession numbers of gene names used in PPI.**

| | |
|---|---|
| CTSBa | PQ683368 |
| CTSBb | PQ683369 |
| CD74a | NP_571665.1 |
| MHC2b | NP_001007207.2 |
| MHC2bl | XP_005167291.1 |
| MHC2dab | NP_571551.3 |
| MHC2dcb | NP_571781.2 |
| CTSD | NP_571785.2 |
| CST3 | NP_001026843.2 |
| PSAP | NP_571958.2 |
| BIDa | NP_001073295.1 |
| LOC571282 | XP_699949.2 |
| LOC100332583 | XP_002665954.4 |
| zgc:153067 | NP_001070245.1 |
| si:zfos-2070c2.3 | XP_017207468.1 |
| LOC791723 | NP_001092896.1 |
| zgc:153129 | AAI24646.1 |
| zgc:162351 | NP_001083019.2 |
| zgc:65811 | NP_956846.1 |
| zgc:66382 | NP_955899.2 |

**Table S4. Abbreviations of gene names used in PPI.**

| | |
|---|---|
| CTSBa | cathepsin Ba |
| CTSBb | cathepsin Bb |
| BIDa | BH3 interacting domain death agonist |
| CD74a | CD74 molecule, major histocompatibility complex, class II invariant chain a |
| CST3 | cystatin C (amyloid angiopathy and cerebral hemorrhage) |
| CTSD | cathepsin D |
| LOC100332583 | toll-like receptor 8 |
| LOC571282 | major histocompatibility complex class II d8.46a |
| LOC791723 | major histocompatibility complex class II DAA gene |
| MHC2b | major histocompatibility complex class II DGB gene |
| MHC2bl | MHC class II beta chain-like |
| MHC2dab | major histocompatibility complex class II DAB gene |
| MHC2dcb | major histocompatibility complex class II DCB gene |
| PSAP | prosaposin |
| si: zfos-2070c2.3 | sucrase-isomaltase |
| zgc:153067 | major histocompatibility complex class II DBB gene |
| zgc:153129 | cystatin 14b, tandem duplicate 1 |
| zgc:162351 | major histocompatibility complex class I ZEA |
| zgc:65811 | zgc:65811 |
| zgc:66382 | serine protease 59, tandem duplicate 1 |

**Table S5. Abbreviations of gene names and accession numbers used for sequence identification.**

| Abbreviation | Accession number |
|---|---|
| CTSB Atlantic salmon | XP_014060893.1 |
| CTSB Chicken | NP_990702.3 |
| CTSB Fugu | XP_003971767.2 |
| CTSB Mouse | NP_031824.1 |
| CTSB Human | NP_001304166.1 |
| CTSB Medaka | XP_020555295.2 |
| CTSB Tilapia | XP_003454569.1 |
| CTSBb Rainbow trout | NP_001117776.1 |
| CTSB Tongue sole | XP_008311144.1 |
| CTSBa Zebrafish | NP_998501.1 |
